# Editorial: Big Data – The Language and Future of One Medicine, One Science

**DOI:** 10.3389/fvets.2018.00114

**Published:** 2018-05-31

**Authors:** Andres M. Perez

**Affiliations:** Department of Veterinary Population Medicine, College of Veterinary Medicine, University of Minnesota, Saint Paul, MN, United States

**Keywords:** Big Data, veterinary epidemiology, disease prevention, disease control, diagnosis

## Introduction

The volume and complexity of data available to support disease animal production, and disease prevention and control, has dramatically grown over the last decade. Such rapid increase in the quantity of data has not necessarily resulted in a consequent ability to improve the quality of management and policy decisions applied to health and production (1). Consequently, there is an urgent need for increasing our ability to apply computational tools to mine big agro-ecosystems data through an interdisciplinary team of agricultural, medical and social scientists, in order to improve efficiency of food production systems and provide science-based inputs for management and policy, with the ultimate objective of improving health and well-being of local and global communities.

The research topic here includes five scientific contributions emerging from presentations, and subsequent debate and discussion, offered at a workshop on Big Data-related research applied to disease prevention and control under the umbrella of one medicine, one science. The workshop was held as part of the international Conference on One Medicine, One Science (iCOMOS) in Minnesota, April 2016, which is one of the leading conferences in the field. The research topic reviews the present and future of Big Data analysis applied to veterinary medicine, providing some theoretical background to build the foundations of the discipline, and demonstrating its application to veterinary sciences in the fields of veterinary epidemiology and animal health care.

## Basic principles and concepts

VanderWaal et al. explored the increasing availability and complexity of data, which has led to emerging opportunities and challenges in veterinary epidemiology. Most of those challenges and opportunities relate to need for translating abundant, diverse, and rapidly growing data into science-based decisions applied to animal health management and policy. The authors argue that Big Data analytics may be used to understand health risks and mitigate the impact of animal disease through identifying high-risk populations, combining data or processes acting at multiple scales through epidemiological modeling approaches, and harnessing high velocity data to monitor animal health trends and detect emerging health threats. The authors also anticipate the need for incorporating novel analytical techniques into the field of veterinary epidemiology, including, for example, machine learning and coding and establishing pipelines to analyze Big Data in near real-time. Ultimately, there is a need for transforming “Big Data” into “smart data,” to support the design and implementation of effective and efficient management and policy decisions to prevent and mitigate the impact of animal disease.

## Big Data analysis applied to veterinary epidemiology

Durr et al. illustrated the application of Big Data analytical approaches to disease spread prediction modeling reassessing a series of papers published between 1977 and 1991 intended to elucidate the probability that outbreaks of bluetongue (BT) and African horse sickness (AHS) might have occurred via long distance wind dispersion (LDWD) of the disease insect vector (Culicoides spp.). Durr et al. used publicly accessible climate data-sets of atmospheric conditions to reassess six of the scenarios described in those early contributions, but allowing for the dispersal of individual midges, modelled as particles, from the purported sources. A custom-built Big Data application (“TAPPAS”) which couples a user-friendly web-interface with an established atmospheric dispersal model (“HYSPLIT”) was used for the simulations. For the two AHS outbreaks (Cyprus 1960 and Spain 1966) there was strong support of the role of LDWD, but for the BT outbreaks, the reassessments were more complex. Except for the western Turkey example, the reanalysis shows the limitation of LDWD modeling when used by itself, and indicates the need for the integration of susceptible host population distribution (and other covariate) data into the modeling process. Results presented here suggest that Big Data analysis, which would allow for the incorporation of large and complex datasets into the analytical pipeline, will have an increasingly important role in providing validation for inferred LDWD models.

Vilalta et al. introduced results of the analysis of a large dataset, referred to as the Swine Health Monitoring Project (SHMP), on Porcine Reproductive and Respiratory Syndrome (PRRS), which is a disease that causes substantial losses in North America. The SHMP is a voluntary program in which sow farm PRRS status is shared weekly by program participants. Analytic tools applied to SHMP-data included spatial, temporal, and phylogenetic analyses that have helped to elucidate the cyclical patterns of PRRS, described the impact that emerging pathogens have had on that pattern, identified its relation with environmental factors, such as precipitation or land cover, identified PRRSv emerging strains, and assessed the influence that voluntary reporting has had on disease control. The value that the research added to practitioners and producers activities is an example of the impact that Big Data analysis may have in practice, helping to create the foundations for a near real-time prediction of disease. The authors argue that implementation of a surveillance system and routine analysis of Big Data, even in the absence of a regulatory framework, may help in the prevention and control of the most devastating disease affecting the North American swine industry.

## Big Data analysis applied to veterinary healthcare

Advances in high-throughput molecular biology and electronic health records, coupled with increasing computer capabilities have also resulted in an increased interest in the use of Big Data in healthcare. McCue and McCoy argue that the collection and analysis of data at an unprecedented scale is leading to a paradigm shift in healthcare because knowledge will be generated faster, data collection and analysis will be less biased, and the biology and pathophysiologic processes underlying disease occurrence will be better understood, compared to traditional scientific approaches. Data would be collected from numerous, disparate sources, at cellular, individual, and population levels, and it is expected that the analysis of those data would lead to personalized disease prognosis, and implementation of precision medicine procedures for patients, with improved accuracy because treatments would be related to an individual's unique combination of genes, environmental risk, and precise disease phenotype. Collection and relation of data from electronic health records across countries and organizations will give access to patient data on a scale previously unimaginable, allowing for precise phenotype definition and objective evaluation of risk factors and response to therapy, and exploring new molecular pathophysiology and disease etiology mechanisms. Additionally, such integration may also help to assess the relations between human and animal diseases supporting control and prevention of diseases at the One Health interface.

Muellner et al. provide an example of a prototype to capture data from electronic medical records for use in national surveillance systems and clinical research in New Zealand. Clinical data and diagnoses from a subset of 344 patient consults were extracted from two veterinary clinics and incorporated into a dedicated database to be analyzed at the population level. Companion animal and equine veterinary practitioners were asked to test the feasibility of this national practice-based health information and data system, and to identify strategies that would increase practitioners' engagement. The geospatial integration of data, for example, would help to establish the baseline incidence of companion animal and equine disease in New Zealand, detect anomalies that may suggest an emerging disease threat or welfare issue, improve disease management, support research activities, and inform policy. Results demonstrated, however, the need for a high degree of usability and smart interface design to scale-up the prototype into a near-real time system for the country.

## Final remarks

In summary, the research topic here provides an overview of the theoretical concepts underlying the application of Big Data analysis to veterinary science and medicine, providing a few examples of implementation of related approaches to epidemiology and health care. Application of Big Data analysis to management and policy in practice is still on its infancy, and the opportunities are not exempt of challenges, including the need for multidisciplinary work in the development of the field, the development of required infrastructure to scale-up its application, and the education of a disciplinary workforce with the necessary knowledge and skills to implement the technology in the field.

## Author contributions

The author confirms being the sole contributor of this work and approved it for publication.

### Conflict of interest statement

The author declares that the research was conducted in the absence of any commercial or financial relationships that could be construed as a potential conflict of interest.
